# Assessment of Knowledge, Attitude and Practices of the Hospital and Community Pharmacists in Saudi Arabia (Jeddah) towards Inappropriate Medication Use in Older Adults

**DOI:** 10.3390/ijerph20021635

**Published:** 2023-01-16

**Authors:** Adel A. Alfahmi, Colin M. Curtain, Mohammed S. Salahudeen

**Affiliations:** School of Pharmacy and Pharmacology, College of Health and Medicine, University of Tasmania, Hobart 7000, Australia

**Keywords:** potentially inappropriate medication, PIMs, Beers criteria, older adults, STOPP/START, adverse drug reactions, pharmacists

## Abstract

In Saudi Arabia, the older adult population is growing and is projected to increase three-fold by 2030. Potentially inappropriate medications (PIMs) are harmful to older adults’ and have a direct impact on clinical, health and economic outcomes. Pharmacists have a vital role in medication tailoring for older adults as multidisciplinary team members. However, there is also a paucity of research regarding pharmacists’ participation in detecting and avoiding PIMs use among older adults in Saudi Arabia. A cross-sectional, self-administered survey was conducted to assess the knowledge, practices, and attitude of pharmacists from seven hospitals and ten community pharmacies in Jeddah, Saudi Arabia. The survey comprised three sections; (i) identifying participants’ general characteristics, (ii) assessing their knowledge of PIMs use in older adults and (iii) examining the pharmacist’s attitude towards the procedures followed in dispensing for older adults. Inferential and descriptive statistics were used to analyse the survey data. A total of 157 community and hospital pharmacists participated in this study. Most of them dispensed medication weekly to older adults (85.4%), and 43.3% had relevant work experience of six to ten years. Though 44.6% of the participants were aware of PIMs that older adults should avoid, only 10.8% claimed adequate knowledge about PIMs. From the given three clinical case scenarios, a minority of pharmacists (21.7%) chose the correct answers, with a mean score of 2.38 ± 2.91 (95% CI 2.35–3.15). Participants who claimed to have knowledge of PIMs had a significantly higher mean score than those who did not, 4.59 ± 2.81 25 (95% CI 2.35–2.61). A minority of the pharmacists (14.7%) used screening tools such as STOPP, Beers criteria, or Medication Appropriateness Index (MAI) to detect PIMs in older adults. No statistically significant differences were detected when comparing the levels of knowledge of pharmacists with 1 to 5 years of practice to pharmacists with 6 to 15 and more years of experience (*p =* 0.431). Pharmacists’ knowledge, attitude and practices toward PIMs use in older adults in Saudi Arabia should be improved. The application of PIMs detection tools such as STOPP/START or Beers criteria currently has no place in day-to-day pharmacists’ roles in Saudi Arabia. Therefore, concerned stakeholders should develop educational programs to improve pharmacists’ knowledge of PIMs and promote the effective use of PIM screening tools such as Beers and STOPP criteria in their practice.

## 1. Introduction

Globally, the population of older persons aged 60 years and older is growing much faster compared to other age groups [1], and is expected to exceed two billion in 2050 [1]. In Saudi Arabia, 3.5% of the total population was aged 65 and above in 2014, which is projected to increase three-fold by 2030 [2]. People aged 65 and above are at a higher risk of drug-related problems (DRPs) [2]. Medication management in older adults can be complicated due to factors such as multiple diseases, complex medication regimes and the ageing process [3]. These factors make older adults more susceptible to adverse drug events (ADEs) that heighten sensitivity to the therapeutic impacts [4,5].

Polypharmacy (use of five or more medicines) is related to an increased risk of potentially inappropriate medication (PIM) use [6], drug duplication, drug-drug interactions, medication non-adherence, and ADEs, leading to poor health outcomes and an increased financial burden to the healthcare system [7,8,9,10]. PIM is defined as a medication that should be avoided in older adults due to their considerable risk of adverse reactions and the lack of clinical evidence behind their benefits when safer and more or equally effective therapeutic options are available [11]. PIM use occurs when the risk associated with the prescribed drugs outweighs potential benefits, especially when effective alternatives are available [12] whereas, ADEs are considered the fifth most common cause of mortality among hospitalised patients [13,14]. Several studies reported the cause of most ADEs as PIM, which are common in ambulatory care [15].

The prevalence of PIM use among older adults was investigated extensively worldwide and in different settings (hospital, aged care, and community), particularly in Western countries [16,17,18,19,20]. A recent systemic review of studies from 11 countries revealed one in five prescriptions provided to older patients in primary care are inappropriate [21]. A nationwide study among older adults in Qatar reported 62.6% had been exposed to PIMs [22]. In Saudi Arabia, Jastaniah et al. reported that 72.6% of the 135 patients included in the study were prescribed PIMs, and 27.4% used only one medication [23]. Harrison et al. found that 81.4% of patients in Australia who had been exposed to a PIM [24] with a higher total medication cost were associated with PIM use [24]. The lack of physicians’ and pharmacists’ knowledge about PIMs, its consequences and the use of prescribing guidelines such as STOPP/START or Beers criteria is a major concern in many countries, including Saudi Arabia [25]. Some researchers suggested that physicians’ formal education about identifying and avoiding PIM utilisation might correlate with improved prescribing in older adults [26,27]. However, others believe that appropriate prescribing for older adults should be in the context of a multidisciplinary team that includes a pharmacist [28].

There is a paucity of evidence regarding the pharmacist’s role in detecting and preventing PIM use among older adults in Saudi Arabia. This challenge has created the need for a national health survey in this regard. Hence, this study explored the pharmacists’ knowledge, attitude and practice regarding the existing explicit and implicit tools available to detect and prevent PIMs use in older adults (e.g., Beers, STOPP/START, FORTA or Medication Appropriateness Index (MAI) criteria). In addition, the study assessed pharmacists’ knowledge and attitude regarding the potential impact of PIM use on older adults’ health and the benefits of avoiding or substituting them with a suitable alternative.

## 2. Methods

### 2.1. Setting and Study Design

A cross-sectional self-administered survey questionnaire was developed after reviewing the literature and conducted over six months from March 2021 to August 2021 [26,29]. The survey addressed the following research questions:

(1) Were pharmacists aware of any PIM tools?
(2) Were they using any criteria in practice to identify PIMs?
(3) Whether pharmacists usually advise physicians about PIMs?
(4) If the pharmacists are unaware of the PIM tools, what was their opinion on implementing them in practice?

### 2.2. Survey Development

The survey questionnaire’s face and content validity was appraised by two independent reviewers and also by the Tasmanian Health and Medical Human Research Ethics Committee at the University of Tasmania, Australia. Before commencing the survey, a pilot investigation was conducted, including ten experienced hospital pharmacists from two targeted hospitals to validate the study. The pilot run ensured that for each participant, the survey took approximately 10 min to complete.

The survey was in English (English is the standard language used in the Saudi health care system) and consisted of three sections:

Section A: Demographics of participants.

Section B: Knowledge and practice of PIM use in older people and the existing criteria used to identify them.

Section C: Pharmacists’ attitudes towards the procedures they follow in filling in older adults’ prescriptions.

We assessed pharmacists’ knowledge of PIMs with the aid of STOPP/START and 2019 Beers criteria within three clinical case scenarios. The three vignettes were developed similarly to that previously utilised by Ravishankar Ramaswamy et al. [26]. The first question of the three-case scenarios has two PIMs, while the rest of the questions have only one. To get a score for the correct answer the participant must choose all PIMs. The total number of correct answers for all participants was used to obtain the knowledge score; however, the study did not set a cut-off score to assess the level of knowledge. The third section was assessed using a 5-point Likert scale ranging from ‘1 = strongly agree’ to ‘5 = strongly disagree’. An information sheet explaining the purpose of the survey was attached to the questionnaire. The survey also had a free-text question on participants’ opinions on implementing a tool to identify PIM use in current practice.

### 2.3. Identifying and Recruiting Participants for the Research

Inclusion criteria: Only registered pharmacists in Saudi Arabia currently working in the hospital, or community pharmacy dispensing were deemed eligible to participate.

Exclusion criteria: Any participants who were not willing to provide informed consent or submitted an incomplete survey were excluded from the study.

According to the recent Saudi census, the total number of pharmacists in Saudi Arabia was 29,125 in 2018 [30,31]. As determined by the sample size calculator (Raosoft^®^ online calculator—http://www.raosoft.com/samplesize.html, accessed on 16 January 2021), the minimum sample size was 380, with 95% CI and a 5% margin of error was estimated for a national-level study. However, limiting the study location to a single city (Jeddah, Saudi Arabia), we anticipate a sample size of 200 participants [32]. As the research included a particular sub-set of people, purposive sampling was employed due to the participants’ characteristics being pharmacists. While selecting hospital or community pharmacists with various demographic traits, such as the type and location of the pharmacy they worked in, a purposive sampling technique seems beneficial. Pharmacists who worked in major public hospitals and community pharmacies in Jeddah were selected. With the permission of the Ministry of Health Pharmaceutical Care Administration (Jeddah, Saudi Arabia branch), the study description, inclusion criteria, and contact information of the researcher were emailed on the 3 March 2021 to pharmacy managers of seven major hospitals and ten community pharmacies in Jeddah, Saudi Arabia. In turn, pharmacy managers emailed all pharmacists in their pharmacy/department about this study. Pharmacists who fit the inclusion criteria and were interested in joining the study contacted the researcher directly. In addition, we provided a flyer with a brief description of the study, and the inclusion criteria were posted in all pharmaceutical care departments in seven major hospitals and ten community pharmacies in Jeddah, Saudi Arabia. We obtained written consent from the participants involved in this study. The main reason for choosing these hospitals is the presence of many experienced pharmacists and the fact that they provide health care to a large number of older adults. The community pharmacies were selected because of their busy location in Jeddah city, serving a substantial percentage of older populations in their areas. By including pharmacists from selected hospitals and community pharmacies in this study, we anticipate a broad coverage of all geographical regions within Jeddah city and ensure our survey sample’s diversity.

### 2.4. Data Analysis

After survey completion, each survey was entered and analysed using IBM SPSS software (version 28, IBM Corp., Armonk, NY, USA). Variables were summarized descriptively using mean and standard deviation median and range or count and percentage. Comparisons between groups were made according to the type of variable. Wilcoxon rank-sum test (Mann–Whitney U test) and a t-test were conducted to compare demographics and profession-related variables. Information on knowledge and practice were reported descriptively. Data (demographics, professional information, and responses to questions regarding attitude) from the hospital and community pharmacy participants were compared and evaluated using Pearson’s Chi-square test for categorical variables and a t-test for continuous variables. The level of significance was set at <0.05.

### 2.5. Ethics

The first author (AA) was a Master’s research student at the University of Tasmania, Australia, and therefore, we sought approval from the Tasmanian Health and Medical Human Research Ethics Committee to undertake this study (reference number H0018582). As well, we obtained approval from the Ministry of Health Medical Research Ethics Committee in Saudi Arabia (Research and Studies Centre/Directorate of Health Affairs Jeddah).

## 3. Results

A total of two hundred hospital and community pharmacists were approached to participate in the study. After excluding those who provided incomplete responses and declined to participate, 157 questionnaires were used for the data analysis, yielding a response rate of 78.5%. The demographics of the respondents and their awareness of PIMs used in older adults are summarised in Table 1. The majority of the participants were Saudi nationals (77.1%), with a bachelor’s degree (65.6%), and worked in hospitals (71.3%). The mean period of practice was 9.7 ± 4.8 (49%) years, with 0.6%, 31.2%, 43.3%, 17.8%, and 7% of pharmacists having worked for less than a year, 1 to 5 years, 6–10 years, 11 to 15 years, and more than 15 years, respectively. Most respondents stated that 100 to 300 (40.8%) of their patients were sixty-five and above each week, while 49.7% mentioned that 25 to 50% of their patients were elderly.

Pharmacists with knowledge of PIMs constituted 44.6%. Though 44.6% of the participants were aware of PIMs that older adults should avoid, only 10.8% claimed adequate knowledge about PIMs. Interestingly, more than a quarter (34.4%) of the participants did not advise physicians about PIM use in older adults while performing a medication review (Table 1). Others (24.5%) said they do inform physicians about PIMs; however, some responded (19.8%) that they provide this information to physicians, but not all the time, while (20.4%) preferred not to answer. Around 33.1% of participants attributed the lack of knowledge among pharmacists to the absence of guidelines and references. In comparison, 23.6% considered the non-existence of communications between healthcare professionals as a relevant factor. Some pharmacists connected this matter to the lack of time (10.2%) or lack of pharmacists’ commitment (14.7%) (Table 1).

There is a considerable gap in Saudi pharmacists’ PIM knowledge, compared to non-Saudis (5.2, *p* = 0.023). Only 39.7% of Saudi nationals were aware of PIM use in older adults, compared to non-Saudi pharmacists (61.1%). Of the pharmacists who claimed knowledge of PIMs, only 38.8% rated their level of knowledge as good, while 61.1% considered it inadequate. There were significant differences in the pharmacists’ awareness of PIMs to be avoided by older adults, considering the work field (60.0% of community pharmacists were aware vs. 38.4% of hospital pharmacists, 6.1, *p* = 0.014). However, no association was found between ethnicity and level of knowledge of PIMs between Saudi and non-Saudi pharmacists (Table 2).

Based on the three case scenarios, pharmacists’ knowledge had a mean score of 2.38 ± 2.91 (95% CI 2.35–3.15), and the majority demonstrated sufficient knowledge by answering the correct options (Table 3). Built on participants’ level of education, no significant differences were detected in total knowledge scores. Participants with knowledge of PIMs showed significantly (*p* =0.05) higher mean scores than those who did not know PIMs, 4.59 ± 2.81 (95% CI 2.35–2.61). Work experience did not show any effect on the mean knowledge score, as the highest level of knowledge was noted in pharmacists with 6–10 years of work experience (11.76%). Pharmacists’ awareness of PIMs was significantly higher in community pharmacies (60.0%) than in hospitals (38.4% *p* = 0.014). More hospital pharmacists exhibited poor knowledge of PIMs than community pharmacists (66.0% and 48.9%, respectively, *p* = 0.046).

Years of experience did not affect the pharmacists’ awareness and knowledge of PIMs in older adults. No statistically significant differences were detected when comparing pharmacists’ knowledge levels with 1 to 5 years of practice to pharmacists with 6 to 15 years of experience (*p =* 0.431). In addition, the study showed no statistically significant differences in the pharmacist’s awareness of PIMs in older adults between the two groups (*p* = 0.431). Moreover, work experience did not reflect on the pharmacists’ knowledge in the three case scenarios (*p* = 0.917, *p* = 0.491, *p* = 0.990).

PIMs were described by 37.6% of the participants as medications in which the risk of an adverse event outweighs their clinical benefit when there is a safer or more effective alternative therapy for the same condition. At the same time, there was a conflict of opinion regarding the purpose of using PIM tools such as Beers or STOPP criteria, with 18.5% of the participants considering the purpose as the detection and avoidance of PIM use in older adults. Others (14.7%) saw the purpose as determining the medications to be used with caution in older adults. However, most pharmacists (67.5%) did not know the purpose of these tools.

Less than half of the pharmacists (40.1%) identified PIM use in older people with the help of medical sources to confirm. Some pharmacists (1.3%) felt no need for tools like Beers or STOPP criteria to identify PIMs to guide prescribing decisions, as it was solely the physician’s role. When participants were asked about the medical resources they use as a guide in older adults’ medication review, 93.6% indicated that they used none. However, seven pharmacists used either STOPP (4.5%), MAI (3.8%), Beers criteria (3.8%), STRIP (1.91%), FORTA (0.6%), PRISCUS list (0.6%) or APAC (0.6%) in their practice.

Our results show medical textbooks are the primary source for detecting PIM use in older adults, according to 74.5% of the pharmacists, followed by online journal articles (36.9%), asking pharmacy colleagues (14.0%), and checking with prescribing physicians (10.8%). Interestingly, 21.0% of participants were not using any references such as textbooks, websites, manuals, guidelines, or hospital protocols to detect PIMs.

Most pharmacists strongly supported the idea of having PIM tools in their practice and provided some qualitative feedback. One pharmacist said, “*the medical field requires these tools to improve medication use for older adults*.” Another pharmacist stated, “*using this kind of tool is going to make pharmacist’s life much easier by detecting medication error and drug-related problems in an effective and timely manner*.” However, not all pharmacists agreed. “*I don’t think that implementing these tools is helpful to pharmacists*”, a pharmacist said, pleading insufficient time during the busy day, while another felt that “*these tools are part of the doctor’s job and there is no need to involve pharmacists in them*”.

Pharmacists reported that they always check patients’ age (94.9%), medication history (91.7%), drug-disease interactions (91%), renal and hepatic functions (91.7%), and drug-drug interactions (93.7%). Only 2.5% always used tools such as Beers criteria and STOPP, when evaluating older adults’ medications. Moreover, 14.7% of pharmacists who participated in this study considered PIM tools as essential in prescribing for older adults (Table 4).

## 4. Discussion

Overall, the knowledge levels of pharmacists in this study showed a compelling need for a PIM education program. The pharmacists’ level of knowledge of PIMs was reported to be below average, however in contrast, the majority answered correctly with the three clinical scenarios. Most of the participants (55%) had poor awareness about PIMs, with less than a quarter obtaining a high score in the case scenario assessments. Our study showed a poor awareness (55%) of PIMs tools compared to a Malaysian study conducted among 277 pharmacists (27%) [33].

Pharmacists working at the outpatient hospital pharmacy were expected to be more knowledgeable of PIMs compared to community pharmacists, due to their convenience in checking medical notes and dealing with a significant number of older patients with multiple comorbidities and medications. Nevertheless, community pharmacists who participated in this study showed a better understanding of PIMs. This poor awareness is alarming and requires the attention of stakeholders (e.g., Saudi Commission for Health Specialties and Saudi Pharmacy Education providers) as similar studies reported that pharmacists were keen to improve their knowledge and clinical skills in providing optimal healthcare for older adults [34,35,36,37].

Despite having poor knowledge and awareness about PIM screening tools, respondents showed a high degree of self-confidence in detecting PIMs in older adults. Our findings align with a similar study from Palestine, which showed that 34% of their participants had poor awareness of the PIM tool [38]. Surprisingly, half the participants were confident enough to identify PIMs by utilising the appropriate medication references. However, most hospital and community pharmacists were unaware of PIM screening tools. Even a higher level of education or work experience did not affect pharmacists’ awareness of PIMs or screening tools. Notwithstanding their poor awareness of PIMs, most pharmacists showed good practice behaviour when dispensing medications to older adults.

Ammerman et al. explained that many healthcare professionals are often tentative in deprescribing a medication provided by other healthcare professionals [39]. However, they concluded that clinical pharmacists have a more significant role in influencing the deprescribing decision because they are trained medication experts who can provide education and documentation of the risk and benefits of PIMs [39]. Most clinical pharmacists in Saudi Arabia hold postgraduate residency training (postgraduate year one (PGY1)), or specialized training (postgraduate year two PGY2)), in domestic or international training programs. On an institutional level, clinical pharmacists in Saudi Arabia are involved in managing cardiology, ambulatory care, and anticoagulation clinics. They work under a collaborative agreement with doctors that permits pharmacists to prescribe medications and order laboratory tests as long as they adhere to the agreement’s terms and conditions. Presently, there is no national regulation that supports any official prescribing by pharmacists in Saudi Arabia, these actions are nonetheless regarded as informal forms of prescribing [40]. Zehnder et al. stated that there is a demand for more websites tailored to pharmacists’ needs and suggested that pharmacists have to quickly adapt to the changes in drug information resources ahead to remain competitive [41]. In Saudi Arabia, PIM use among older adults is a significant challenge for healthcare professionals that may compromise their efforts in advancing older adults’ health [42]. There is also a paucity of data regarding the pharmacists’ role in detecting and preventing PIM use among older adults in Saudi Arabia. Some pharmacists attributed their inability to perform PIM reviews to the lack of time.

To some extent, most healthcare professionals agree that executing such a critical task requires more free time for pharmacists. Therefore, pharmacists’ time could be freed by shifting dispensing tasks to pharmacy technicians and dealing with more complex medication management problems and reconciliation. Other barriers may affect pharmacists’ role and limit their positive intervention in the deprescribing decisions. Lau et al. concluded that there is empirical evidence for pharmacist involvement in medication reviews, improving prescribing practices and reducing PIMs use among older adults [43]. They anticipated that specific barriers associated with resources and environmental context (limited access to pharmacists, time constraints, lack of electronic clinical decision support systems) might be more prominent in this setting [43].

Collaboration between physicians and pharmacists can lead to better treatment outcomes for older adults. Pharmacists should be encouraged to communicate with physicians when necessary. Alsuhebany et al. found that the lack of constant understanding of the clinical pharmacist role in Saudi Arabia’s health system leads to communication defects and affects their participation level with multidisciplinary teams [44]. Only a quarter of the participants disclosed advising physicians about PIMs in older adults. We found that one in five pharmacists preferred not to answer the question about advising physicians against PIM use in older adults. From their perspective, this could be due to the sensitive nature of this question, as some pharmacists find it challenging to communicate with physicians. Clinical pharmacy practice is newly introduced in the Saudi Arabian health system, and few hospitals approve and support clinical pharmacy practice [45].

Since 16.6% of hospital admissions of older adults occur due to ADRs caused by PIMs use [46], PIMs deprescribing can lead to a decrease in the risk of PIMs adverse outcomes (such as falls, hospital admissions, and premature mortality) [47,48]. These could be important to encourage pharmacists to improve their deprescribing decision-making role. There is a strong demand to create educational awareness of PIMs in older adults among practising pharmacists in Saudi Arabia. The lack of understanding of PIM tools among Saudi pharmacists reflects the gap in the pharmacy education system, particularly among new graduates. A comprehensive initiative for highlighting the critical role of hospital and community pharmacists in detecting PIMs would be beneficial. Formal education programs for undergraduate pharmacy students and continuous professional development for practising pharmacy are also necessary. Pharmacists could benefit from adding PIMs knowledge to their workplace’s ongoing medical education programs.

## 5. Strengths and Limitations

Our study on PIMs use in Saudi Arabia is quite unique, considering a dearth of similar studies across the Middle East. To our knowledge, this is the first study conducted in Saudi Arabia to measure hospital and community pharmacists’ knowledge, attitude and practices toward PIMs use in older adults. This study distinguishes itself from other literature in its focus on pharmacists working in Saudi Arabia. On top of that, the study had a well-designed survey questionnaire that an expert panel has evaluated. On the other side, the survey was conducted in seven government hospitals’ outpatient pharmacies, and ten community pharmacies in Jeddah, Saudi Arabia, with reasonable sample size and may not generalise/reflect the reality among pharmacists in Saudi Arabia. We were unable to conduct an online survey due to cultural issues; therefore, paper surveys were our method of choice. The paper survey distribution was conducted during the COVID-19 pandemic, which significantly impacted the study’s progress due to the government’s curfew during the outbreak and the difficulty of entering hospitals with the newly implemented COVID-19 protocol. The study relied on self-reporting, which may not suggest factual practice, given the knowledge scores in this study indicate that pharmacists have poor knowledge of PIMs in older adults. Moreover, the study represents the opinions of 157 out of the 380 needed pharmacists, thus while the findings can be regarded as significant and instructive, however they do not necessarily reflect the views of all Saudi pharmacists. A larger national-level study is mandated to see the representation of pharmacist views across different states.

## 6. Conclusions

The knowledge, attitude, and practices toward PIMs use in older adults were conducted among 157 hospital and community pharmacists in Jeddah, Saudi Arabia. Pharmacists with knowledge of PIMs constituted 44.6%, of which 10.8% claimed adequate knowledge about PIMs. The results of this study revealed the necessity of integrating a standardised PIM tool into the healthcare system. We recommended targeted drug safety education programs about identifying PIM use in older adults during undergraduate and postgraduate university degrees along with continuing professional development (CPD) training for practising pharmacists. In conclusion, with an upsurge in the ageing population and the clinical, health and economic consequences of PIMs, a nationwide educational intervention relating to PIMs can be implemented as a strategy to improve quality use of medicine among older adults.

## Figures and Tables

**Table 1:** Characteristics of the study population (N = 157).

Characteristics	Participants n (%)
**Ethnicity**	
Saudi Arabian	121 (77.1)
Non-Saudi	36 (22.9)
**Level of Pharmacy Education**	
Diploma	41 (26.1)
Bachelors	103 (65.6)
Masters	11 (7.0)
PhD	2 (1.27)
**Working Field**	
Hospital	112 (71.3)
Community	45 (28.7)
**Work Experience**	
Less than 1 year	1 (0.6)
1–5 years	49 (31.2)
6–10 years	68 (43.3)
11–15 years	28 (17.8)
More than 15 years	11 (7.0)
**Number of patients older than 65 seen by pharmacists per week**	
Less than 50	19 (12.1)
50–100	46 (29.3)
100–300	64 (40.8)
300–600	23 (14.7)
**Percentage of older adults seen by pharmacists**	
Less than 25%	23 (14.7)
25 to 50%	78 (49.7)
50 to 75%	56 (35.7)
Above 75%	0 (0.0)
**Pharmacists with prior knowledge about PIMs**	
Yes	70 (44.6)
No	87 (55.4)
**PIMs tools employed as a guide in older adults prescribing**	
None	147 (93.6)
STOPP	7 (4.5)
FORTA	1 (0.6)
MAI	6 (3.8)
Beers Criteria	3 (1.9)
The PRISCUS list	1 (0.6)
APAC	1 (0.6)
STRIP	3 (1.9)
**Pharmacists’ knowledge of PIMs in older adults**	
Poor	96 (61.1)
Adequate	44 (28.0)
Good	17 (10.8)
**Pharmacists advised physicians about PIMs**	
Yes	31 (19.8)
No	54 (34.4)
Sometimes	40 (25.5)
Not applicable	32 (20.4)
**Reason for increasing concerns about PIMs use among older adults**	
Lack of communication among healthcare professionals.	37 (23.6)
Lack of resources (e.g., guidelines, references etc.).	52 (33.1)
Lack of research on PIMs use in older adults.	50 (31.9)
Lack of education regarding PIMs use in older adults.	106 (67.5)
Lack of knowledge among healthcare professionals regarding PIMs and detection tools.	131 (83.4)
Lack of time.	16 (10.2)
Lack of pharmacist commitment.	23 (14.7)
Other.	6 (3.8)

PIMs, Potentially Inappropriate Medications; STOPP, Screening tool of older people’s prescriptions; FORTA, The Fit fOR The Aged classification system; MAI, Medication Appropriateness Index; Beers Criteria for Potentially Inappropriate Medication Use in Older Patients; PRISCUS, German list called PRISCUS (Latin for “old and venerable”); APAC, Australian prescribing appropriateness criteria; PhD, Doctor of Philosophy.

**Table 2:** Pharmacists’ knowledge and awareness about PIMs with the consideration of ethnicity and work field.

Characteristics	Awareness of PIMs		*p*-Value	Level of Knowledge		*p*-Value
	Yes	No		Poor	Good	
Saudi	48 (39.7%)	73 (83.9%)	0.023	79 (65.29%)	42 (34.72%)	0.051
Non-Saudi	22 (61.1%)	14 (16.1%)		17 (47.22%)	19 (52.78%)	
Hospital	43 (38.4%)	69 (61.6%)	0.014	74 (66.0%)	38 (33.9%)	0.046
Community	27 (60.0%)	18 (40.0%)		22 (48.9%)	23 (51.1%)	

PIMs, Potentially Inappropriate Medications.

**Table 3:** Assessing pharmacists’ knowledge of PIM use based on clinical case scenarios.

Mrs AM is 70 years old with Diabetes Mellitus, hypertension and urinary incontinence. To prevent her urinary incontinence from being exacerbated, which medication would you like to avoid (indicate multiple options can be selected)? * Correct response: Prazosin, Donepezil	
Oxybutynin	6 (3.8%)
Prazosin *	99 (63.0%)
Fluoxetine	17 (10.8%)
Donepezil *	75 (47.8%)
Paracetamol	4 (2.6%)
I don’t know	25 (15.9%)
Which one of the following antihypertensive drugs would you like to remove from Mr TH’s medication list? He is 68 years old, and his blood pressure is under control with no other medical conditions. * Correct response: Clonidine.	
Perindopril	7 (4.5%)
Amlodipine	22 (14.0%)
Clonidine *	91 (58.0%)
Hydrochlorothiazide	15 (9.6%)
I don’t know	22 (14.0%)
Mrs SM is an 80-year-old female newly diagnosed with Parkinson’s disease who comes to your pharmacy, asking you something for her nausea. Which of the following would you recommend? * Correct response: Domperidone.	
Domperidone *	72 (45.9%)
Promethazine	6 (3.8%)
Metoclopramide	54 (34.4%)
Prochlorperazine	2 (1.3%)
I don’t know	23 (14.7%)

**Table 4:** Pharmacists’ attitude towards the procedures to follow when dispensing prescriptions for older adults.

Pharmacist’s Attitude-Based Questions	Agree n (%)	Neutral n (%)	Disagree n (%)
I always check the patients’ age	149 (94.9)	8 (5.1)	0(0.0)
Checking patients’ medication history is an important role that pharmacists play	144 (91.7)	12 (7.6)	1 (0.6)
I frequently check for drug-disease interactions	143 (91.0)	14 (8.9)	0 (0.0)
I frequently check renal and hepatic function before dispensing medications	144 (91.7)	12 (7.6)	1 (0.6)
I regularly look for drug-drug interactions	247 (93.7)	10 (6.4)	0 (0.0)
I always use tools such as Beers criteria, STOPP when evaluating older adults’ medications	4 (2.5)	23 (14.7)	130 (70.4)
The use of PIMs tools is essential in prescribing for older adults	23 (14.7)	54 (34.4)	80 (51.0)

PIM = potentially inappropriate medication.

## Data Availability

The datasets generated and/or analysed during the current study are available from the corresponding author upon reasonable request.
